# ExoPD-L1: an assistant for tumor progression and potential diagnostic marker

**DOI:** 10.3389/fonc.2023.1194180

**Published:** 2023-09-05

**Authors:** Rong Hu, Md Shoykot Jahan, Lijun Tang

**Affiliations:** ^1^ School of Life Sciences, Central South University, Changsha, China; ^2^ Xiangya School of Medicine, Central South University, Changsha, China

**Keywords:** ExoPD-L1, immunosuppression, regulatory factors, ExoPD-L1 quantification, combination therapy

## Abstract

The proliferation and function of immune cells are often inhibited by the binding of programmed cell-death ligand 1 (PD-L1) to programmed cell-death 1 (PD-1). So far, many studies have shown that this combination poses significant difficulties for cancer treatment. Fortunately, PD-L1/PD-1 blocking therapy has achieved satisfactory results. Exosomes are tiny extracellular vesicle particles with a diameter of 40~160 nm, formed by cells through endocytosis. The exosomes are a natural shelter for many molecules and an important medium for information transmission. The contents of exosomes are composed of DNA, RNA, proteins and lipids etc. They are crucial to antigen presentation, tumor invasion, cell differentiation and migration. In addition to being present on the surface of tumor cells or in soluble form, PD-L1 is carried into the extracellular environment by tumor derived exosomes (TEX). At this time, the exosomes serve as a medium for communication between tumor cells and other cells or tissues and organs. In this review, we will cover the immunosuppressive role of exosomal PD-L1 (ExoPD-L1), ExoPD-L1 regulatory factors and emerging approaches for quantifying and detecting ExoPD-L1. More importantly, we will discuss how targeted ExoPD-L1 and combination therapy can be used to treat cancer more effectively.

## 1 Introduction

An immune checkpoint protein called Programmed cell-death ligand 1 (PD-L1, B7-H1, CD274) has been found in various cells, including tumor cells and some immune cells (1, 2). In the immunosuppressive mechanisms, PD-L1/PD-1 plays a crucial role. When PD-L1 interacts with PD-1 on T cells, the intracellular protein tyrosine phosphatase SHP-2 activates the downstream signal pathway of PD-1, which impedes T-cell activation, that prevents T cells from killing cancer cells by preventing the proliferation and function of T-cells (3, 4). Currently, immune checkpoint inhibitors (PD-L1 or PD-1 monoclonal antibodies) have been utilized to treat various cancers (5). However, researchers are focusing on exosomes because they carry a variety of protein biomarkers linked with a particular disease and hence have the potential to function as early-stage disease diagnostic tools (6).

Exosomes are vesicles of about 40~160nm (on average 100nm) formed by endocytosis and can be secreted by variety of cells. Various molecules are contained within them, including DNA, RNA, protein, lipid and metabolites. Exosomes transport antigens and communicates between cells (7–9). It has been proven that exosomes are found in a wide range of biological fluids, including serum, plasma, breast milk, urine, cerebrospinal fluid, tears, bile and so forth (10, 11). Nevertheless, exosomes do not contain glycolytic enzymes and cytoskeleton proteins, and the release of active DNA does not rely on small extracellular vesicles as carriers. In addition, extracellular vesicles, including exosomes, do not transport double-stranded DNA and DNA-binding histones (12). The classification of exosomes is regulated by primitive cells, but the mechanism remains unclear. Their composition and surface markers reflect their origin. Therefore, in addition to the general exosomal markers that help distinguish exosomes from other extracellular vesicles (such as CD81, CD9, CD63, ALIX and TSG101), it can also detect exosomes based on special membrane surface markers from different cell sources. In addition, exosome lipid content plays a significant role in preserving the shapes of exosomes, participating in biogenesis and regulating the homeostasis of recipient cells (7, 12, 13). Exosomes are closely linked to the immune system’s activation, immunosuppression and immune escape (14). It was reported that PD-L1 exists in the exosomes of melanoma cell lines SK-MEL-28, B16F10, human lung cancer cell lines A549 and human embryonic lung fibroblast cell lineMRC-5 (15).

**Figure 1:**
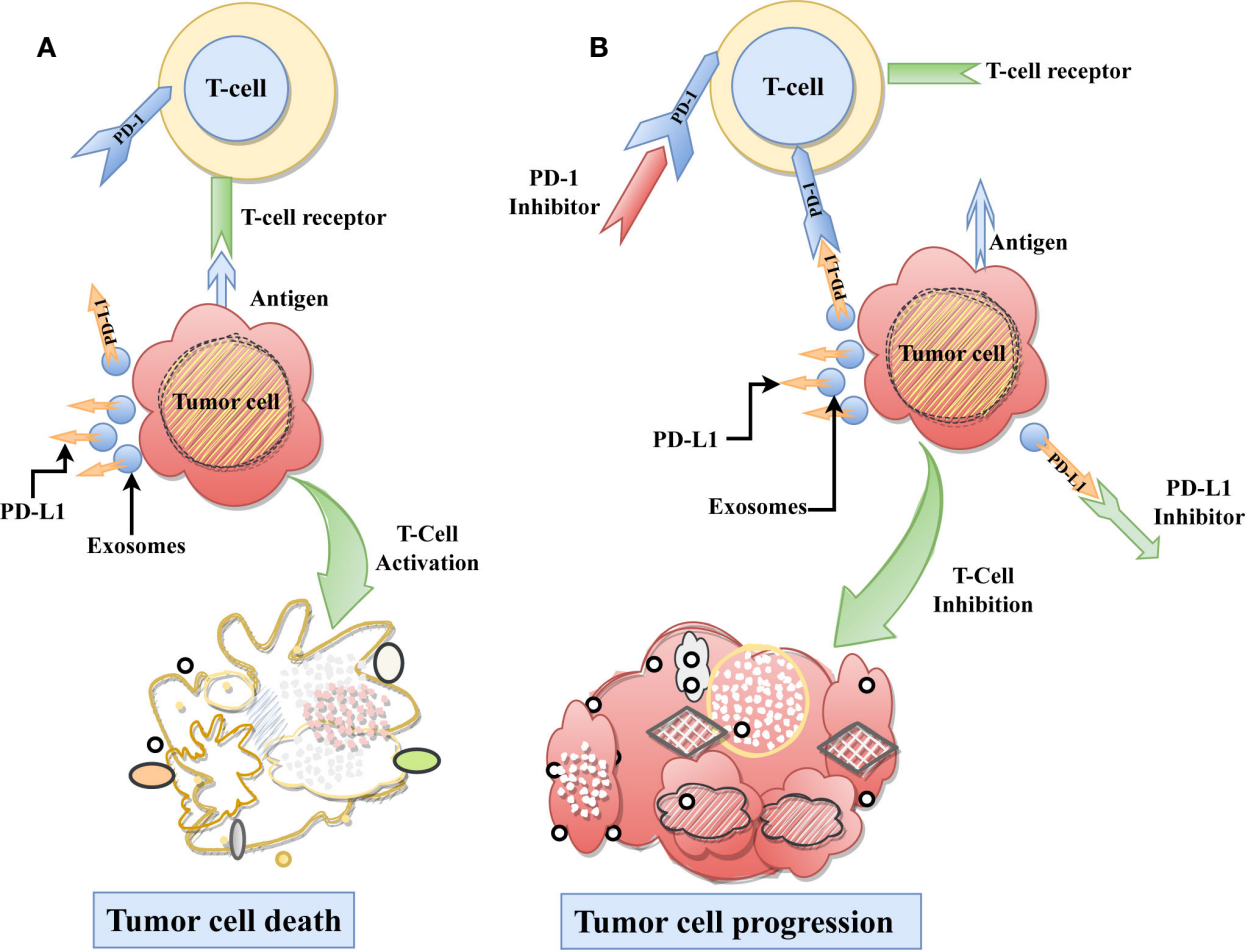
Role ExoPD-L1 in Immunosuppression. **(A)** As part of the typical immunological process, T-cells recognize the tumor-specific antigen and subsequently destroying the tumor cell. **(B)** On the other hand, ExoPD-L1 derived from tumor cells, causes T cell dysfunction by binding with the PD-1 receptor found on T-cells, which ultimately results in the progression of tumor cells. Moreover, using immune checkpoint inhibitors on the PD-L1 or PD-1 receptors can be restored immune-mediated tumor control and inhibit tumor growth.

Exosomal PD-L1 (ExoPD-L1), like other forms of PD-L1 in cells, impedes immune cell function and promotes tumor development (16). ExoPD-L1 suppresses T-cell activities by attaching to PD-1 on the T-cell surface. PD-1 is a security checkpoint molecule that assists in preventing the immune system from attacking healthy cells and tissues. ExoPD-L1 attaches to PD-1 and sends a signal to T-cells, regulating their activity and suppressing the immunological response, leading to cancer cell proliferation. Conversely, PD-1 or PD-L1 inhibitors can be used to halt tumor growth by blocking their activity (**Figure 1**). Thus it is crucial to understand how ExoPD-L1 contributes to cancer development and the different regulatory factors affecting the expression of ExoPD-L1. In this review, we highlight the role of ExoPD-L1 in immunosuppression, the factors that cause its upregulation, its utility as a marker for cancer diagnosis, detection methods for ExoPD-L1, and its potential as a target for combination therapy in cancer treatment.

## 2 ExoPD-L1 promotes tumor progression through the PD-L1/PD-1 axis

Research has uncovered the presence of ExoPD-L1 in various cancer cell lines (7). Therefore, exosomal PD/PD-L1 is assumed to be an intersection of inflammation and tumor progression so, it is necessary to investigate the causing factors and mechanisms that lead to T-cell dysfunction.

### 2.1 ExoPD-L1 expression in tumor cells

PD-L1 can be upregulated in cancerous and in non-cancerous cells, such as macrophages, monocytes, and tumor cells. Besides, PD-1 receptors are expressed on most activated CD4^+^ and CD8^+^ T-cells, monocytes, B-cells, and MDSCs (17). PD-L1/PD-1 interaction allows tumor cells to escape immune surveillance by suppressing T-cell growth and function (18). A variety of factors lead to tumor cells expressing PD-L1. For instance, constitutive oncogenic signaling pathways, epigenetic modifications, genomic aberrations, and different tumor microenvironment associated factors such as proinflammatory signaling and hypoxia (19). An association was found between ExoPD-L1 and the ESCRT protein ALIX in breast cancer. Depletion of ALIX decreases ExoPD-L1 release and enhances PD-L1 expression on the cell surface, leading to an upregulation of oncogenic signaling mediated by epidermal growth factor receptor (EGFR) activity. Based on this, the ESCRT-accessory protein known as ALIX regulates the distribution of PD-L1 across cell and exosome membranes. This implies that ALIX expression and PD-L1 expression are inversely related (20). Another study showed that removing both nSMase2 and Rab27a from the PC3 prostate cancer cells prevented the release of ExoPD-L1. Additionally, lowering nSMase2 activity decreased PD-L1 transcription (21). It is believed that ExoPD-L1 expression has a corresponding immunosuppressive mechanism in response to interferon-gamma (IFN-γ), which is produced by CD8^+^ cytotoxic T cells, dendritic cells (DCs), macrophages, and Natural killer (NK) cells (22). A study demonstrated the link between IFN-γ and the overexpression of ExoPD-L1. It was found that exosomes derived from glioblastoma contain PD-L1, which binds to PD-1 and inhibits the activation of T cells (23). In addition, glioblastoma-derived exosomes contain various protein components associated with the EIF2, eIF4/mTOR, ephrin receptor, and IGF-1 signaling pathways. These associated proteins upregulate PD-L1 expression and are correlated with the increased phosphorylation of STAT3 and ERK1/2 in monocytes, thus inhibiting T cell tumor infiltration (24). A study by Kim et al., found that plasma ExoPD-L1 levels were associated with PD-L1-positive tumors in patients with non-small-cell lung cancer (NSCLC). When PD-L1 was present in the exosomes of Jurkat T cells, IFN-γ secretion was suppressed. In contrast, IFN-γ production was restored when PD-L1 was inhibited or eliminated from the exosomes (25). These findings suggest a link between ExoPD-L1 and various cancers. In order to develop novel therapeutic agents, it is crucial first to understand what drives ExoPD-L1 upregulation. Thus blocking these molecules using inhibitors may have therapeutic utility in cancer immunotherapy.

### 2.2 ExoPD-L1 promotes tumor progression by inhibiting T cell function

The evidence suggests that ExoPD-L1 plays a crucial role in progression of breast cancer (26), colorectal cancer, prostate cancer (21), non-small cell lung cancer (25, 27), gastric cancer (28, 29), osteosarcoma (30), pancreatic cancer (31), head and neck cancer (32), glioblastoma (33). A previous study found that, when T cells were exposed to tumor-derived exosomes (TEXs), the lymphocyte mRNA expression levels were changed depending on doses, cell type and cell activation. TEXs down-regulated adenosine-pathway genes which, resulted in elevated levels of CD39 expression and increased adenosine synthesis (34). Yang et al. found that ExoPD-L1 significantly suppresses CD3/CD28 induced ERK phosphorylation of T cells and NF-kB activation. Moreover ExoPD-L1 can also reduce the T cell activation factors, PHA-induced interleukin-2 (IL-2) and Granzyme B secretion (26). High levels of ExoPD-L1 in colorectal liver metastasis patients reduce the infiltration and activation of T lymphocyte, and ExoPD-L1 have promising prognostic values in colorectal liver metastasis patients (35). Moreover, the level of ExoPD-L1 in gastric cancer patients has also been found to be negatively associated with the number of CD8^+^ T and CD4^+^ T cells, as well as with CD69 (a T-cell activation marker) and PD-1 of CD8^+^ effector T cells. MHC and T cell receptor(TCR) must bind together for T cell activation, but ExoPD-L1 acts as a co-inhibitor, leading to T cell dysfunction (28). Additionally, increasing ExoPD-L1 with activated CD8^+^ effector T cells decreased CD69 of T cells in NSCLC (32). In both prostate and colorectal cancer models, ExoPD-L1 inhibited T cells activation in draining lymph nodes and reduced CD8^+^/CD4^+^ T cells proportions (21).

### 2.3 ExoPD-L1 facilitates the establishment of pre-metastatic niche to promote tumor metastasis

Exosomes play a significant role during the exchange of information between primary tumors and distant organs. In addition, they can package tumor information with proteins and nucleic acids and transport it to distant organs. This is crucial for the formation of a pre-metastatic niche (9), which plays a key role in tumor metastasis and growth. TEXs contain immunosuppressive molecules and immune-inhibiting factors. Their effects also include impairment of immune cell production, development, maturation and activity, as well as the apoptosis of immune cells and abnormal differentiation of some stem cells (36). It was found that the exosomes from bone marrow-derived cells (BMDCs) inhibited the CD8^+^ T cells proliferation and promoted tumor growth through PD-L1, and may promote tumor metastasis through ExoPD-L1 (37). The most common factors associated with tumor pre-metastatic niches are immunosuppression, inflammation, angiogenesis and vascular permeability, lymph angiogenesis, organ formation and reprogramming (38). Exosomes derived from tumor cells are heterogeneous. Exosomes play a positive as well as a negative role in the establishment of tumor pre-metastasis niche. On the one hand exosomes produced by non-metastatic tumors inhibit tumor metastasis by enhancing NK cell recruitment and influencing macrophage polarization (39). On the other hand, they facilitate tumor metastasis by forming a pro-inflammatory environment (40), regulating proliferation, adhesion, immunosuppression (41), promoting angiogenesis and vascular permeability (42). Evidence shows that metastatic tumor exosomes contain higher levels of PD-L1 than primary tumor exosomes, suggesting that ExoPD-L1 plays a role in pre-metastatic niche establishment (43).

## 3 Hidden influencer driving increased exosomal PD-L1 expression

It is believed that multiple mechanisms are involved in regulating PD-L1 expression in cells. It includes the following aspects: various oncogenic pathways of the tumor microenvironment, transcription factors and regulation of PD-L1 at post-transcriptional regulation level (different miRNAs) (44–47). The type 3 transmembrane protein CMTM 6 is widely expressed in organisms. Through co-localizing with intracellular PD-L1, CMTM6 reduced PD-L1 ubiquitination to reduce PD-L1 degradation by lysosomes, thereby enhancing PD-L1 inhibitory ability on T cells (48, 49). Another member of the CMTM family, CMTM4, also contributes to PD-L1 expression. It was found that PD-L1 was more effectively suppressed by inhibiting both CMTM4 and CMTM6 simultaneously (49). In recent years, research has focused on the RNA binding protein (RBP) in order to understand PD-L1 regulation. Human antigen R as an RBP binds to the 3’UTR of CMTM6 mRNA that is highly enriched in AU elements (AREs), resulting in more stable CMTM6 mRNA, thereby inducing PD-L1 expression (50). Tu et al. developed a PD-L1 antibody called H1A. Compared to earlier PD-L1 antibodies, it has a distinct mechanism. H1A mainly destroys the binding of CMTM6 and PD-L1, making PD-L1 more susceptible to lysosomal degradation (1). Tristetraprolin, as RBP, is phosphorylated upon oncogenic RAS signaling, reducing its ability to bind to mRNA, thereby reducing its inhibitory effect on PD-L1 (51). Apart from PD-L1 inhibiting immune function by connecting to PD-1, PD-L1 can also be used as an RBP to compete with SC10 and SC4 components of RNA exosome for DNA repair elements NBS1 and BRCA1 mRNA, protecting target RNA from degradation and enhancing resistance to DNA damage. PD-L1 is up-regulated after DNA damage, which is effective for the immune escape of tumor cells and resistance to DNA damage treatment.

The ExoPD-L1 expression has been found to strongly correlate with transforming growth factor (TGF-β) in the tumor microenvironment in breast cancer cells. They revealed that TGF-β increased the ExoPD-L1, which impaired CD8^+^ T cell function. Furthermore, combining exosome loss and TGF-β inhibition enhanced T cell function (52). According to reports, when T cells are activated, they secrete IFN-γ, which could up-regulate ExoPD-L1 and then ExoPD-L1 inhibits T cell’s function, promoting tumor growth. However, it was found that ExoPD-L1 expression is significantly linked with IFN-γ in patients with metastatic melanoma, and ExoPD-L1 inhibits CD8^+^ T cell functions. They also observed that ExoPD-L1 levels were positively linked with IFN-γ and tumor load in patients and that ExoPD-L1 concentration varied during anti-PD-1 treatment (43). In human papillomavirus positive cervical squamous cell carcinoma cell lines SiHa and CaSki showed overexpression of CXCL10 binds to CXCR3, which is highly expressed in human foreskin fibroblasts activates JAK-STAT pathway, which up-regulates PD-L1 and ExoPD-L1 (53). Another study also showed that the level of ExoPD-L1 in metastatic melanoma was higher than that in healthy people and primary melanoma, but the number of exosomes was not notably different (43).

5-Fluorouracil (5-FU) down-regulated the expression of cytotoxic factors IFN-γ, TNF-α, IL-2, IL-6 and GM-CSF in gastric cancer and up-regulated the expression of PD-L1 through the miR-940/Cbl-b/STAT5A axis. At the same time, it significantly up-regulated the expression of ExoPD-L1 (54). Sulfasalazine (SAS) is an FDA-approved cystine/glutamate antiporter xCT inhibitor. The study found that the level of glutamate in the plasma of patients with melanoma increased significantly, which was conducive to tumor growth. SAS inhibited xCT by up-regulating reactive oxygen species, thereby inhibiting tumor progression. However, when combined with xCT inhibition and anti-PD-1/PD-L1 antibody, SAS enhanced the binding of transcription factors IRF4 and EGR1 to PD-L1 promoter, increased PD-L1 mRNA and protein levels, increased tumor ExoPD-L1 level, and reduced CD8^+^ T cell activity (47).

## 4 ExoPD-L1 can be used as a disease diagnostic marker

Due to their characteristics and composition, exosomes are being increasingly used as diagnostic tools. Exosomes reflect their parent cell’s characteristics through their distinctive contents. Exosomes from cancer cells possess distinctive genetic and epigenetic makeup which, makes them tumorigenic (55, 56).

### 4.1 ExoPD-L1 being a reliable marker for cancer tracking

Accessing exosome cargo in biological fluid samples is easy because these complex particles are present in all body fluids. Exosome-based liquid biopsies can potentially be used in the diagnosis and prognosis of cancer. Exosomes carry therapeutic payloads, including antisense oligonucleotides, immunomodulators, therapeutic medicines, and vaccinations. They can also be directed at a particular target (57). Clinical research focuses on exosomes as potential cancer biomarkers due to their abundance and propensity to be identified in circulation (6). The release of exosomes is more prevalent in malignancies, suggesting a possible role in cancer pathophysiology (58). Cell membranes contain PD-L1 as a protein, soluble or encapsulated in exosomes (59–61). ExoPD-L1, a special form of PD-L1, binds to immune cell’s PD-1 and inhibits their proliferation and immune function, thereby promoting tumor growth and metastasis (62). Patients with head and neck cancer were found to have plasma-derived exosomes that carried functional PD-L1, which was related to the clinicopathological characteristics of patients, rather than soluble PD-L1 (sPD-L1) (32). In a study on osteosarcoma, researchers reported that the levels of ExoPD-L1 and N-cadherin in serum were higher than those of healthy people and benign patients. Researchers also found a correlation between PD-L1 concentrations in exosomes and metastasis. They explored that PD-L1 concentrations were notably higher in metastatic osteosarcoma patients than in patients without metastasis. PD-L1 and N-cadherin in exosomes play a vital role in osteosarcoma lung metastasis, so it is feasible to detect their changes in exosomes to predict osteosarcoma lung metastasis progress (30). It has been found by Chen et al. that in hepatocellular carcinoma, Golgi membrane protein 1 promotes the accumulation of PD-L1 in the exosomes by inhibiting Rab27b in the trans-Golgi network area, but does not affect the number of exosomes (63). A second research on melanoma cancer revealed the same findings. ExoPD-L1 was more abundant in the pooled blood of melanoma patients than that of healthy donors, while the quantity of exosomes did not differ significantly (15).

Even though some cancer cells highly express PD-L1, they rarely secrete it into exosomes. The research found that although PD-L1 mRNA was 15 times higher in two prostate cell lines PC3 and DU145 than in melanoma SK-MEL-28, PD-L1 protein levels were similar. Further studies found that these two prostate cancer cells secreted more PD-L1 protein into exosomes, rather than the variation caused by different translation or protein degradation (21). ExoPD-L1 is more easily isolated than membrane PD-L1 and more stable than sPD-L1 (64). More importantly, it’s inhibitory effect on T cells is sometimes more substantial than sPD-L (15, 65). Since PD-L1 expression is heterogeneous and unstable, detecting of PD-L1 level in cells and tissues is often unreliable for disease diagnosis. Exosomes usually exist stably in organisms and can be used as the protector of PD-L1 to prevent it from being degraded by proteases. The detection of the ExoPD-L1 level makes the diagnosis more sensitive and specific. Therefore, it is more reliable to use the ExoPD-L1 level as a diagnostic marker (15, 65).

It is reported by Chen et al. that the PD-L1 level in exosomes indicates the immune status of tumors and is positively associated with disease progression (43). According to another study, PD-L1 positive pancreatic ductal adenocarcinoma patients have a significantly shorter survival time (31). The immunosuppressive ability of exosomes with high PD-L1 is higher than those with low PD-L1 (28). In addition, glycosylated ExoPD-L1 has also been reported as a more reliable tumor diagnostic marker compared to circulating ExoPD-L1 (66). As well, PD-L1 has a negative correlation with the OS of patients with glioblastoma (67), which can be used as a poor prognostic indicator. A link between ExoPD-L1 and OS has been found in gastric cancer (GC). Patients with high ExoPD-L1 had a shorter OS than those with low levels, which could be used as an independent prognostic index for GC patients, whereas sPD-L1 did not have any significant correlation with it (36). Thus, ExoPD-L1 is considered to be a negative prognostic factor.

### 4.2 Methods for detecting and quantifying ExoPD-L1

**Figure 2:**
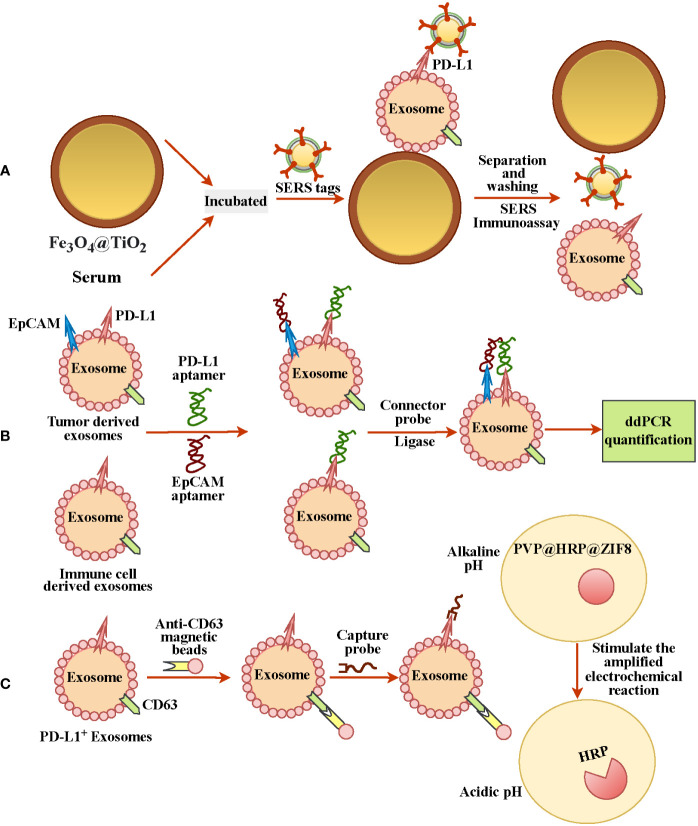
Different methods for detecting and quantifying ExoPD-L1. **(A)** Fe_3_O_4_@TiO_2_ was used to capture ExoPD-L1 in serum, and then PD-L1^+^ exosomes were labeled by SERS tags with anti-PD-L1 antibody for ExoPD-L1 quantification. **(B)** EPCAM tracked exosomes from tumor cells, and adapters of EpCAM and PD-L1 recognized PD-L1^+^ exosomes, and droplet digital PCR detection reflected the number of ExoPD-L1. **(C)** Anti-CD63-functionalized magnetic beads and anti-PD-L1-linked capture probes were used to enrich PD-L1^+^ exosomes. DNA amplification with probes as primers reduced environmental pH and released PD-L1^+^ exosomes recognition by stimulating electrochemical reaction.

Since PD-L1 is expressed heterogeneously in exosomes and exosomes are small, detecting ExoPD-L1 is still a big challenge (68, 69). However, recent studies have reported some effective approaches for extracting exosomes and labeling ExoPD-L1 (**Table 1**). Such as Fe_3_O_4_@TiO_2_ is used to capture exosomes and then combined with an anti-PD-L1 antibody modified surface-enhanced Raman scattering (SERS) tags to label the ExoPD-L1 for quantification; this method has the advantages of low sample size (only 4 µl) and short time (40 mins) (**Figure 2A**) (69). Lin et al. tracked TEXs through the tumor biomarker EpCAM and then recognized TEXs through the aptamers for EpCAM and PD-L1. Since the aptamers have less steric hindrance and membrane fluidity, the two aptamers are close to each other and ligated using connector probes and ligases. In the end, droplet digital PCR, also known as ddPCR, was used to quantify ligation products, which may indicate the quantity of tumor-derived ExoPD-L1 (**Figure 2B**) (70). In recent years, studies have found that glycosylated ExoPD-L1 may be a more reliable marker for diagnosing cancer compared to total ExoPD-L1. Zhu et al. used another natural mannose/glucose binding lectin and PD-L1 glycosylated independent aptamer as probes. When they combine with glycosylated ExoPD-L1 simultaneously, they will also lead to proximity ligation assay (PLA) of probes. The non-glycosylated ExoPD-L1 will not lead to PLA. At this time, the ligation products will be quantified by quantitative real-time PCR to reflect the glycosylated ExoPD-L1 level (66). Previously, they also developed *in situ* imaging methods for exosomal glycosylated proteins, such as ExoPD-L1 glycosylation. This has significant implications for the *in situ* visualization of exosomal protein glycosylation (72). Cao et al. used anti-CD63 functionalized magnetic beads to capture exosomes and then combined them with anti-PD-L1-linked capture probes to enrich PD-L1^+^ exosomes. Then the capture probe was used as the primer for DNA amplification to reduce the environmental pH. At the same time, the author also prepared a pH-responsive protein-encapsulating ZIF-8(PVP@HRP@ZIF8), which is stable under alkaline pH, while horseradish peroxidase (HRP) is released under acidic pH to stimulate the amplified electrochemical reaction, so as to realize the recognition of PD-L1^+^ exosomes (71) (**Figure 2C**).

**Table 1:** The methods for detecting and quantifying ExoPD-L1.

Sample type	Capture exosomes	Detection and quantification of ExoPD-L1	Reference
Serum	Exosomes were captured by Fe3O4@TiO2 nanoparticles	Surface-Enhanced Raman Scattering immunoassay	(69)
Tumor cells	Tumor-derived ExoPD-L1 are recognized by EpCAM and PD-L1 aptamers	Droplet digital PCR	(70)
Tumor cells	The binding of glycosylated ExoPD-L1 to natural mannose/glucose binding lectin and PD-L1 glycosylated independent aptamer probes lead to proximity ligation assay	quantitative real-time PCR	(66)
serum	Exosomes are captured by anti-CD63 functionalized magnetic beads and then bound with anti-PD-L1-linked capture probes to enrich PD-L1^+^ exosomes	pH-responsive protein-encapsulating ZIF-8(PVP@HRP @ZIF8) released horseradish peroxidase at low pH to amplify electrochemical reaction	(71)

In addition, the researchers also developed gold-silver core-shell nano-bipyramids and anti-hPD-L1 antibody-conjugated gold nanorods as two main sensing parts, which can be used to differentiate ExoPD-L1 and sPD-L1, or for ExoPD-L1 analysis, detection and identification of exosome subclasses (73). Qin et al. designed a dual-cycling nanoprobe to quantify PD-L1 and miRNA-21 in exosome lysate simultaneously. The advantages of this method include high sensitivity and low time consumption, as well as the ability to detect circulating ExoPD-L1 and miR-21 in human plasma (74). These ExoPD-L1 isolation, labeling, quantification and detection techniques are of great significance for clinical diagnosis.

## 5 Targeting ExoPD-L1 and combination therapy provide new insights into cancer therapy

Currently, immunotherapy for blocking PD-L1/PD-1 has made remarkable achievements (75). Tumor-derived exosomes are a significant source of PD-L1, and the ExoPD-L1 have a potential role in PD-L1/PD-1 antibody resistance (76). Liu et al. found that PD-L1 expression in melanoma cells could be increased by inhibiting xCT via IRF4/EGR1, thus promoting the secretion of exosome PD-L1. This leads to M2 macrophage polarization and ultimately to the induction of anti-PD-1/PD-L1 therapy resistance (47). Gliblastoma (GBM)-derived stem cells (GSCs) can be enriched by continuous low-dose temozolomide (TMZ) stimulation treatment in GBM. GSCs produced exosomes containing high PD-L1 and interacted with TMZ-sensitive GBM, activated AMPK/ULK1 pathway to promote cell protective autophagy, inhibit cell apoptosis and enhance the TMZ resistance of GBM (33).

Therapies that block the PD-L1/PD-1 pathway have experienced great success in the past, and immunotherapy targeting this pathway has achieved unprecedented, durable response rates in a various cancers (77). Various PD-L1/PD -1 immune checkpoint inhibitors are available in human clinical trials (**Table 2**) for treating multiple tumor types. PD-L1/PD-1 blocking therapy is primarily mediated by tumor-infiltrating T cells (90, 91). However, sometimes using a single method for tumor treatment will only show a weak effect. In addition, researchers found that some PD-L1 tumor patients still responded to PD-L1 monoclonal antibody (mAb) checkpoint inhibitors while receiving anti-PD-L1 mAb treatment. And this is critical for cancer patients treated with immunotherapy (92). Whether PD-L1 positive or negative solid tumor patients, PD-L1/PD-1 inhibitors or antibodies can inhibit tumor growth and prolong the OS of patients. Therefore, the PD-L1 level is not the only basis for selecting inhibitors (37, 93). Researchers have found that combination therapy usually achieves better results. For example, researchers found that the loss of ExoPD-L1 alone has no obvious inhibitory effect on the tumor. Still when the exosome secretion inhibitor GW4869 and ferroptosis inducer (Fe3^+^) were assembled into nano units and combined with an anti-PD-L1 antibody, the results were significant in enhancing T cell activation, proliferation, and in inhibiting melanoma cell metastasis (94). Yang et al. found that when combined with exosome inhibitor GW4869 or knockout Rab27a and PD-L1 antibody, the therapeutic effect of anti-PD-L1 antibody can be enhanced in a PD-L1-dependent manner and much stronger tumor suppression (26). When mice were injected with TRAMP-C2 cells lacking Rab27a and PD-L1, PD-1^+^ CD8^+^ T and CD4^+^ T cells decreased. Therefore, even a brief deletion of ExoPD-L1 will lead to long-term immunity to tumor cells (21). Moreover, in recent years, bispecific antibodies have also been shown to have great advantages in the treatment process. For instance, a bispecific antibody LY3434172, targeting both PD-1 and PD-L1 at the same time, has a stronger activation effect on T cells than using anti-PD-L1 or PD-1 antibody alone or both antibodies at the same time (95). In another study, researchers developed a bispecific antibody, YM101, that binds both TGF-β (a negative regulator of anti-tumor immunity) and PD-L1. This bispecific antibody simultaneously inhibits the biological effects of TGF-β and PD-L1/PD-1 pathways and has more potent anti-tumor effects than anti-TGF-β and anti-PD-L1 monotherapy (96). However, some researchers believe that more attention should be paid to adaptive immune resistance in subsets of cancer patients to better guide cancer immunotherapy, rather than the endless pursuit of combination clinical trials (97). Therefore, either monotherapy or combination clinical trials should benefit patients.

**Table 2:** PD-L1/PD-1 immune checkpoint inhibitors in human clinical trials.

Antibody type	Antibodies name	Notes	Reference
Anti-PD-L1 antibody	Atezolizumab	NCT02425891.A randomized, phase 3 trial. In Triple-Negative Breast Cancer	(78)
		NCT02366143 and NCT02409342. A randomized, open-label, phase 3 trial. In non–small-cell lung cancer	(79, 80)
		NCT03434379. A global, open­label, phase 3 trial. In Hepatocellular Carcinoma	(81)
	Avelumab	NCT02603432. An international, open-label, phase 3 trial. In Urothelial Carcinoma	(82)
		NCT02155647. An international, multicenter, single-arm, open-label, phase IV trial. In Metastatic Merkel Cell Carcinoma	(83)
	Durvalumab	NCT02125461. A multicenter, randomized, double-blind, pla- cebo-controlled, phase 3 trial. In Non-small cell lung cancer	(84)
Anti-PD-1 antibody	Nivolumab	NCT02060188. Patients were enrolled using a Simon two-stage study design. Metastatic Colorectal Cancer	(85)
		NCT02125461. Results from an interim analysis of the randomized, double-blind, international, phase 3 PACIFIC study. In Non-Small-Cell Lung Cancer	(86)
		NCT01844505. A multicenter, randomized, controlled, double-blind, phase 3 trial. In Melanoma	(87)
	Pembrolizumab	NCT01905657. A randomized, controlled, phase 2/3 clinical trial In Non-small cell lung cancer	(88)
		NCT01866319. A randomized, controlled, phase 3 study In Melanoma	(89)

## 6 Conclusion and future perspectives

Evidence has shown ExoPD-L1 promotes tumor progression and metastasis in varieties of cancer cells by suppressing T-cell functions. Moreover, ExoPD-L1 elevations are associated with advanced cancer stages, larger tumor sizes, positive lymph node status, and distant metastasis in NSCLC patients whereas sPD-L1 shows no significant correlation (98). Melanoma patients with ExoPD-L1 have also been shown to have a significant association with tumor response and progression of the disease (15). Multiple variable can up-regulate ExoPD-L1 expression. The IFN-γ and ExoPD-L1 concentrations are correlated positively. When IFN-γ increases ExoPD-L1, it suppresses T-cell activity in metastatic melanoma and glioblastoma (23, 43). In addition, Rab27a and nSMase2 are also responsible for the upregulation of ExoPD-L1 in prostate cancer (21). Therefore, it is essential to identify what triggers the overexpression of PD-L1 in exosomes. Consequently, inhibiting the variables can be beneficial in cancer treatment. Furthermore, it is also crucial to understand the precise molecular mechanism by which ExoPD-L1 suppresses T-cell activity and promotes immunosuppression in cancer cell development. Several inhibitory roles of ExoPD-L1 have been addressed above, the molecular mechanisms involved in ExoPD-L1 mediated immunosuppression are mostly unknown. Therefore, further research is needed to explore the precise mechanism by which ExoPD-L1 suppresses the immune system and promotes tumor growth might be helpful in cancer treatment.

Besides ExoPD-L1, tumor-derived exosomes contain diverse immunomodulators that contribute to tumor progression by impairing immune cell function. However, these molecules can also be noninvasive biomarkers for tracking and treating cancer (36). Exosomal lncRNA RPPH1 enhances colorectal cancer (CRC) metastasis by inducing epithelial-mesenchymal transition (EMT) via interaction with β-tubulin III (TUBB3), blocking its ubiquitination. Exosomal lncRNA RPPH1 levels are closely linked with disease status (99). In a study on breast cancer, TEX containing miR-105 promoted metastasis by disrupting tight junctions in endothelial monolayers. Notably, inhibiting the overexpression of miR-105 in intensely metastatic tumors mitigates these effects (100). TEX-expressing Fas ligand (FasL) suppresses immune function in human and mouse cancer cell lines. They also play a critical role in CD8^+^ T-cell apoptosis; conversely, apoptosis can be encountered by anti-FasL antibodies (101, 102). Tumor-derived extracellular vesicles transfer active TGF-β type II receptors (TβRII) to CD8^+^ T cells, which trigger the activation of SMAD3. This activated SMAD3 associates and cooperated with the TCF1 transcription factor, resulting in CD8^+^ T cell exhaustion, ultimately contributing to immunotherapy failure (103).

In our investigation, we sought a reliable prognostic marker that would enable us to track the course of cancer. The ExoPD-L1 levels are significantly related to the development and response of tumors as well as the survival of patients with metastatic cancer (15, 28, 43). ExoPD-L1 levels tend to rise with tumor progression and are highly connected with overall survival; they can be used as a predictive factor to monitor cancer patients. Moreover, circulating exosomes are more reliable than biopsies and plasma for identifying and quantifying PD-L1. Nevertheless, the size of exosomes and the variability of PD-L1 make it challenging to quantify ExoPD-L1. Thus, it is pivotal to find a precise and simple way to diagnose ExoPD-L1. However, above mentioned effective ExoPD-L1 extraction method (**Figure 2**) can be utilized to diagnose ExoPD-L1. The association of higher ExoPD-L1 expression with cancer suggests that it could also be considered in the cancer treatment by employing PD-1 or PL-L1 inhibitors (**Figure 1**). More importantly, exosome loss should also be considered in developing a combination therapy because generally, the immunosuppression of immune cells by exosomes is PD-L1-dependent.

In a nutshell, the existence of ExoPD-L1 in cancer cells is a hotly debated research area that may have implications for the diagnosis and treatment of cancer. Currently, the research on ExoPD-L1 is still in the infant stage; therefore, further studies are required to clarify the role of ExoPD-L1 in cancer and its potential therapeutic application.

## Author contributions

Study concept and design: RH and LT. Drafting of the manuscript: RH and MJ. Critical revision of the manuscript for important intellectual content: LT and MJ. Obtained funding: RH and LT. All authors contributed to the article and approved the submitted version.
